# Early Failure of Lithium–Sulfur Batteries at Practical Conditions: Crosstalk between Sulfur Cathode and Lithium Anode

**DOI:** 10.1002/advs.202201640

**Published:** 2022-05-07

**Authors:** Lili Shi, Cassidy S. Anderson, Lubhani Mishra, Hong Qiao, Nathan Canfield, Yaobin Xu, Chengqi Wang, TaeJin Jang, Zhaoxin Yu, Shuo Feng, Phung M Le, Venkat R. Subramanian, Chongmin Wang, Jun Liu, Jie Xiao, Dongping Lu

**Affiliations:** ^1^ Energy and Environment Directorate Pacific Northwest National Laboratory Richland WA 99354 USA; ^2^ Walker Department of Mechanical Engineering Texas Materials Institute The University of Texas at Austin Austin TX 78712 USA; ^3^ Environmental Molecular Sciences Laboratory Pacific Northwest National Laboratory Richland WA 99352 USA; ^4^ Department of Nuclear Engineering and Radiological Sciences University of Michigan Ann Arbor MI 48109 USA; ^5^ Materials Science and Engineering Program Texas Materials Institute The University of Texas at Austin Austin TX 78712 USA

**Keywords:** charge failure, internal short circuit, Li–S batteries, surface roughness, topography

## Abstract

Lithium–sulfur (Li–S) batteries are one of the most promising next‐generation energy storage technologies due to their high theoretical energy and low cost. However, Li–S cells with practically high energy still suffer from a very limited cycle life with reasons which remain unclear. Here, through cell study under practical conditions, it is proved that an internal short circuit (ISC) is a root cause of early cell failure and is ascribed to the crosstalk between the S cathode and Li anode. The cathode topography affects S reactions through influencing the local resistance and electrolyte distribution, particularly under lean electrolyte conditions. The inhomogeneous reactions of S cathodes are easily mirrored by the Li anodes, resulting in exaggerated localized Li plating/stripping, Li filament formation, and eventually cell ISC. Manipulating cathode topography is proven effective to extend the cell cycle life under practical conditions. The findings of this work shed new light on the electrode design for extending cycle life of high‐energy Li–S cells, which are also applicable for other rechargeable Li or metal batteries.

## Graphical abstract

An active crosstalk occurs between the cathode and lithium (Li) anode, revealing an important factor influencing cycling of rechargeable Li batteries . The rough cathode surface is copied by the soft Li anode, resulting in inhomogeneous current density and exaggerated localized Li plating/stripping, causing an internal short circuit. Manipulation of cathode topography is required to extend the Li‐cell cycle life.

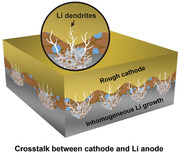

## 1 Introduction

Lithium–sulfur (Li–S) batteries are a promising next‐generation energy storage technology due to the high theoretical specific capacity (1675 mAh g^−1^), low cost, and the innate environmental friendliness of S.^[^ ^1^ ^]^ Despite significant progress made on material development^[^ ^2^ ^]^ and mechanistic understandings,^[^ ^3^ ^]^ the deployment of the Li–S battery technology is still hindered by its low practical energy and limited cycle life.^[^ ^4^ ^]^ In particular, long‐lived Li–S batteries face challenges from both the high mass loading S cathode and deep cycling of the Li anode. For rechargeable batteries employing Li as the anode, its failure mechanisms have been extensively studied and solely interpreted by Li anode problems: 1) the formation of dendritic Li,^[^ ^5^ ^]^ which has a high potential to penetrate the separator and cause an internal short circuit (ISC);^[^ ^6^ ^]^ 2) the Li passivation or “dead” Li, which gets isolated and loses electronic contact as active Li inventory;^[^ ^7^ ^]^ and 3) the electrolyte depletion caused by the continuous interactions with Li.^[^ ^8^ ^]^ Among these causes, Li dendrites not only threaten cell cycling life but also cause safety concerns, which is a bottleneck to maturation and commercialization of Li batteries.^[^ ^9^ ^]^ The Li plating process, which involves Li‐ion mass transfer, charge transfer, and Li nucleation and growth,^[^ ^10^ ^]^ is very complicated and entangled with multiple factors, including electrolyte properties (solvent, salt, and concentration),^[^ ^11^ ^]^ electrochemical reaction parameters (such as current density and potential),^[^ ^5^, ^12^ ^]^ and operational conditions (temperature and pressure).^[^ ^13^ ^]^ Accordingly, many approaches have been proposed for suppressing or eliminating the Li dendrite growth by optimizing electrolyte recipes,^[^ ^14^ ^]^ employing Li‐hosts or Li‐alloy anodes,^[^ ^15^ ^]^ using functional separators,^[^ ^16^ ^]^ or adjusting external pressure and temperature. Promising progress and insightful understandings have been gained from the fundamental studies. Notably, most studies of Li metal focus on either Li|Li symmetric cells or Li‐transition metal oxide (Li/TMO) cells with structure‐stable cathodes. The Li behaviors in Li–S cells are rarely studied due to the complications of S cathodes, which differ from TMO cathodes in terms of material stability and electrode morphology. One significant difference is that S cathodes involve Li‐polysulfide dissolution, diffusion, and redistribution within the cathode and even across the cell during the charging/discharging processes. The spatial redistribution of those S species leads to reactions and morphological variations of cathode upon repeated cycling. Moreover, the S electrode itself is much more porous and rougher than TMO electrodes, which further exaggerates the S redistribution and reaction nonuniformity. Inside a cell, where many layers of cathodes and anodes are stacked together and separated by polymer membrane, the local regions with relatively lower resistance favor the higher reactivity. So, any morphological changes occurring in the S cathodes will eventually affect the local reactions, thereby affecting Li plating/stripping on the counter electrode. Such crosstalk effects between the S cathode and Li anode, particularly under practical conditions, would influence cell cycling life, and have not been studied before. Here, to understand the cell fading mechanism, especially the widely observed early cell failure of high‐energy Li–S batteries, we used patterned electrodes with manipulated surface roughness as example electrodes to study the effects of cathode topography on Li anode and cell cycling. Mitigation approaches are proposed accordingly to extend the cycle life under practical conditions.

## 2 Results and Discussion

**Figure 1:**
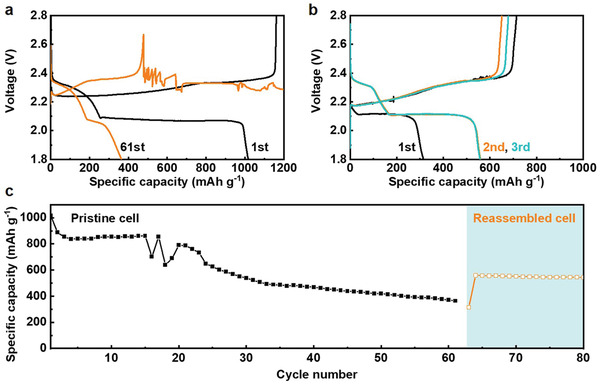
a) The first and last discharge/charge profiles of the BSC electrodes cycled under lean electrolyte conditions (*E*/*S* = 4 mL g^−1^). b) The first three discharge/charge profiles of the reassembled Li–S cell with the cycled S cathode and Li anode harvested from the cell of (a), and with fresh separator and electrolyte (*E*/*S* = 6 mL g^−1^). c) The cycling performance of the pristine and reassembled cells.

To study the cell performance at conditions relevant to practical scenarios, pristine S cathodes without any surface treatment as the baseline S cathodes (BSC) with a high S loading (6 mg cm^−2^) were prepared according to our previous work^[^ ^17^ ^]^ and tested under lean electrolyte conditions (*E*/*S* = 4 mL g^−1^). Typically, the high loading electrodes deliver high initial discharge capacities >1000 mAh g^−1^ at 0.1 C rate with two plateaus at 2.2 and 2.1 V (**Figure** **1**a),^[^ ^18^ ^]^ indicating rational material and electrode structures for efficient S conversion. Upon cycling, the cell capacity remained stable for the first 35 cycles and then began to decay (Figure 1c), mainly because of the polarization increase accompanying S loss and electrolyte depletion.^[^ ^7^, ^19^ ^]^ However, after 60 cycles, the voltage dropped suddenly during charging and fluctuated randomly, failing to reach the cutoff voltage. The endless charging eventually led to an early cell termination, called “charge failure” in this work. Notably, such a failure mode, that is, gradual cycling capacity decay followed by a charge failure, is generally observed in not only high S loading coin cells under both flooded (*E*/*S =* 10 mL g^−1^, Figure S1a,b, Supporting Information) and lean (*E*/*S =* 4 mL g^−1^) electrolyte conditions, but also in pouch cells under practical conditions.^[^ ^19b^ ^]^ This means the charge failure is a common cause of the short life of Li–S cells and should be addressed if pursuing a long lifespan. The well‐known Li polysulfide “shuttling” should be excluded for the charge failure because the observed voltage fluctuations and continuous drops are totally different than the typical “shuttling” behavior, that is, a flat charging plateau at 2.4 V. To understand the charge failure, cell internal resistance (IR) was monitored during the cell cycling. As shown in Figure S1c, Supporting Information, the cell IR experienced a slow increase followed by a sudden drop at the end. The IR increase is due to the electrode passivation and electrolyte depletion. The sudden drop of IR corresponds to quick drop of charging voltage, which suggests an ISC, reducing the overall cell resistance. It should be pointed out that cell IR after ISC event still maintains a value above 0 Ω. This indicates a “soft” ISC which is also supported by observations of voltage curve fluctuations. The failed cells were further disassembled, and the cycled S cathode and Li anode were harvested and reassembled into cells with a new separator and fresh electrolyte. The reassembled cell delivered a limited discharge capacity of 315 mA h g^−1^ in the first discharge (Figure 1b). This agrees well with the failed charging in the last cycle, meaning the cathode is at a partially charged state. In the subsequent cycles, both charge and discharge capacities were recovered to high levels and remained stable. These results indicate that both the cycled S cathode and Li anode remain active, thus materials loss or deactivation is not a cause of the cell failure. Instead, the voltage drop and fluctuation suggest that an ISC or micro ISC is a possible cause of the charge failure and it was studied further.

**Figure 2:**
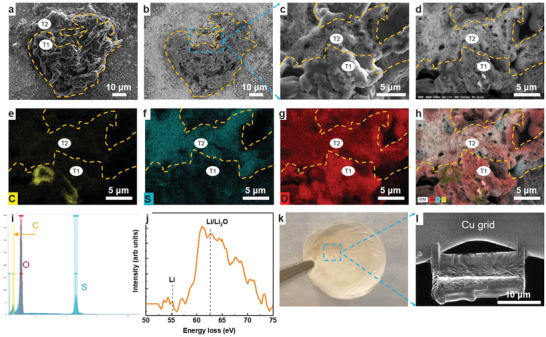
Structural and chemical information of the particles on the separator surface facing cathode (coin cell in Figure 1a after charge failure). a) SEM and b) BSE images of T1 and T2. c,d) Magnified SEM and BSE images of the blue rectangular in (b). e–g) EDX mapping of (c): red—O, blue—S, yellow—C, and h) their combination. i) EDX spectrum of (c). j) EELS of the Li K‐edge on T1. k) Digital photograph of the used separator with the top side facing S cathode. l) SEM image of cross section of particles prepared by focused ion beam (FIB) for EELS.

The separator from the failed cell was harvested and carefully analyzed using scanning electron microscopy (SEM), back‐scattering electron microscopy (BSE), energy‐dispersive X‐ray (EDX) spectroscopy, and electron energy loss spectroscopy (EELS). The cycled separator was washed with ether solvents to remove any soluble polysulfides and lithium salts prior to the characterization. The side of separator facing the cathode was analyzed as the area of interest (**Figure** **2**k). Particles with two types of morphologies were observed on the separator using SEM analysis (Figure 2a). One was composed of large chunks (>5 µm) with smooth edges and surfaces (T1 in Figure 2a). The second type of particle was porous and covered by many small flakes (<1 µm) (T2 in Figure 2a). Clearer differences of those two morphologies were identified by BSE mode SEM as shown in Figure 2b–d, where T1 has a much darker phase contrast than T2 (Figure 2b) under the same testing conditions. This reflects differences in the chemical composition of those two types of morphologies. Since BSE is more sensitive to atomic weight, that is, lighter materials have a darker color, the components of T1 should be lighter than those of T2. The high‐resolution SEM and BSE analyses further confirm the difference and boundaries between T1 and T2 morphologies (Figure 2c,d). Chemical information was further revealed by EDX mapping (Figure 2e–i). The flakes of T2 were made up of S and oxygen (O) elements, which originate from Li‐polysulfides or Li_2_S/Li_2_S_2_ deposits.^[^ ^20^ ^]^ It is noted that O could come from either the residual electrolyte or be caused by air exposure during sample transfer for SEM/EDX analysis. In sharp contrast to T2, the T1 contained a large amount of O and trace amounts of carbon (C) and S, with clear boundaries identified by EDX mapping (Figure 2f,h). The presence of only trace amounts of S excludes the presence of Li‐polysulfides or Li_2_S/Li_2_S_2_ deposits in T1. Similar results were observed on other T1‐like particles (Figure S2, Supporting Information). Li cannot be identified using EDX mapping because of its low atomic weight. However, the dark contrast in BSE and absence of other cations in EDX mapping suggest that the T1 particles are Li metals and Li oxides. Electrolyte decomposition products are possible sources of Li oxides but can be excluded here because no clear fluorine (F) or nitrogen (N) was observed in the area. Further characterization of the T1 by Cryogen (scanning) transmission electron microscope [(S)TEM)]/EELS proves the existence of Li metal inside the particles (Figure 2j). The observation of Li/Li oxides stuck on the separator side facing the S cathode confirms the formation of Li dendrite and its penetration through the separator.

**Figure 3:**
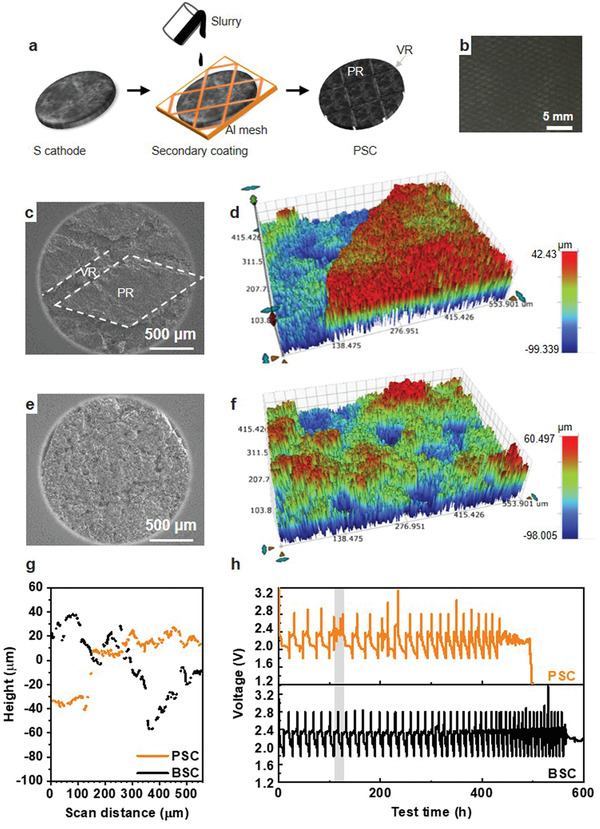
a) Schematic illustration of PSC preparation. b) Digital photograph of the PSC, SEM, and surface profilometric images of c,d) PSC and e,f) BSC. The color from blue to red represents the height from low to high. g) *X* line‐scan profiles of PSC and BSC. h) Discharge and charge profiles of the PSC and BSC upon cycling at an *E*/*S* of 4 mL g^−1^. The grey bar highlights the early occurrence of ISC in PSC.

It is well‐known that Li growth is affected by the local current density,^[^ ^21^ ^]^ Li‐ion concentration gradient,^[^ ^22^ ^]^ electrolyte mass transfer,^[^ ^23^ ^]^ etc. Thus, any change in these factors would affect the morphologies of the Li plating. To form a complete circuit loop in cell operation, equivalent charge transfer should happen on both the cathode and anode, simultaneously, while the proneness of the reactions depends on the local resistance between the two electrodes. For a given electrode chemistry, any factors affecting local resistance may cause variation in local reactions. This is particularly true in S electrodes, which are not only rough and porous, but also involve S species redistribution. To study the impact of the cathode on Li growth, the patterned S cathodes (PSC) with patterned surfaces were prepared to represent the amplified rough surface. The PSC with a S loading of 6 mg cm^−2^ was prepared by applying a double‐layer coating using aluminum (Al) mesh as a template (**Figure** **3**a). Copying the Al mesh template, the PSC showed diamond‐shaped peak regions (PR) and linear valley regions (VR) (Figure 3b,c). The optical profilometry measurement indicates that the PR resides ≈60 µm higher than the VR, and the average roughness of the whole electrode is ≈25 µm (Figure 3d,g; Figure S3a, Table S1, Supporting Information). For comparison, the BSC without any patterns has an overall flatter surface (Figure 3e) and an average surface roughness of 20 µm (Figure 3f). The BSC and PSC were assembled into coin cells and tested under lean electrolyte conditions (*E*/*S* = 4 mL g^−1^) (Figure 3h). As expected, the BSC showed high specific capacity and a long cycle life under the lean electrolyte conditions. The charge failure occurred after 563 h. However, in the PSC cell, the charge failure occurred much earlier during the fourth cycle (70 h), meaning an early occurrence of ISC (highlighted in grey in Figure 3h). The cell was recovered after a prolonged and fluctuated charge cycle, indicating the occurrence of a micro or soft ISC.^[^ ^24^ ^]^ Despite the recovery, the cell failed after 303 h of cycling. Increasing the electrolyte amount (*E*/*S* ratio) helped extend the cycle life for both electrodes (Figure S3b–e, Supporting Information), but the trend in charge failure, that is, PSC had much shorter cycle life than BSC, remained the same. This further confirms the prevailing role of electrode topography in cell cycling, where a rougher surface leads to quicker formation of Li dendrites, and thus earlier cell failure.

**Figure 4:**
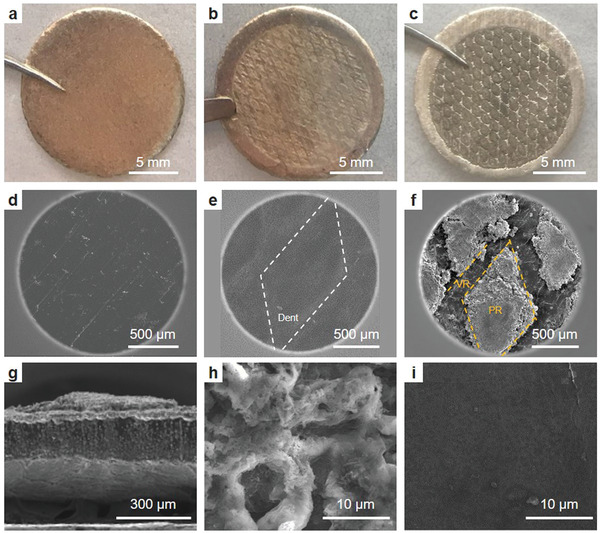
Digital photographs (a–c) and SEM images (d–i) of the pristine a,d) Li anode, b,e) the Li anode after assembly but before cycling, and c,f–i) the Li anode after cycling with PSC. g) Cross section of (f). Higher‐resolution images of h) PR and i) VR of (f).

To understand the effects of cathode topography on Li electrodeposition, the Li anodes before and after cycling in the PSC cells were analyzed. Li anode morphology was studied and compared at three scenarios: 1) the pristine Li anode before assembly, 2) after assembly into a coin cell with the PSC before cycling, and 3) after cycling with the PSC. The pristine Li anode showed a flat and smooth surface (**Figure** **4**a,d). After cell assembly with the PSC, the cathode morphology imprinted onto the Li anode, showing diamond dents and raised linear surfaces. This means that the PR of the cathode have better contact with Li metal due to the higher local pressure (Figure 4b,e). After cycling, the Li from the diamond dents transformed into diamond PR (Figure 4c,f,g), suggesting extensive reactions and volume expansion. SEM characterizations indicated the PR were very porous and composed of entangled powders and wires (Figure 4h), while the linear VR remained flat and dense as the pristine Li (Figure 4i). The chemical compositions of the PR and VR were further analyzed by XPS (Figure S4, Supporting Information). More C, O, and S species were identified in PR than in the VR. These results suggest that because of the better contact, the Li with more compact contact with the cathode participated more in the Li striping/plating and thus in more side reactions with the electrolyte, thereby generating significant volume expansion.

**Figure 5:**
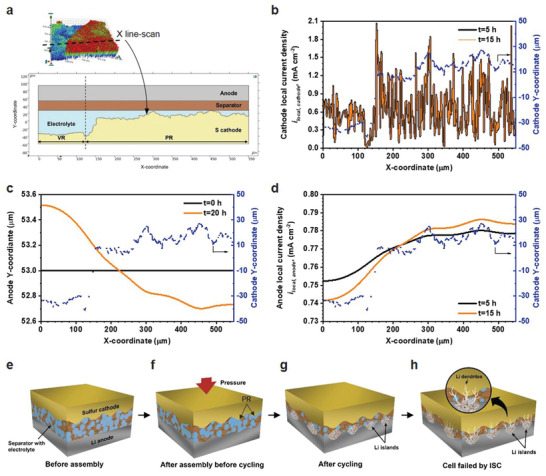
Simulation and schematic illustration of the *i*_local_ distribution and Li anode evolution in the Li–S cell with rough cathode. a) Geometry of the model. b) *i*_local, cathode_ distribution. d) *i*_local, anode_ distribution in the first discharge (*t* = 5 h) and charge (*t* = 15 h). *t* = 0–10 h is the first discharge. *t* = 10–20 h is the first charge. c) The moving boundary of Li anode at the beginning (*t* = 0 h) and the end of first cycle (*t* = 20 h). e) The rough cathode, separator, and Li metal anode before they are assembled in a cell. f) The rough cathode imprints its pattern on the soft Li metal anode. g) During cycling, Li islands form in the PR protruding from Li metal anode. h) Sharp Li metal dendrites penetrate through the separator causing an ISC.

To understand the local current density (*i*_local_) distribution and Li anode evolution when working with a rough cathode, 2D Li–S cells including mass and charge transport were simulated (**Figure** **5** and Supporting Information). A part of PSC containing both VR and PR (Figure 3g) was extracted and used as the cathode topography (Figure 5a). First, a simplified model was used to study the effect of cathode topography where the cathode and anode were assumed as reaction surfaces.^[^ ^26^, ^27^, ^28^, ^29^ ^]^ Only Li^+^ and anion (TFSI^−^) were assumed as the dissolved species in the electrolyte. The Li^+^ reaction kinetics is determined by the ion diffusion and electromigration.^[^ ^25^ ^]^ When the diffusion rate is small, the electromigration plays the leading role. The distance between the cathode and anode affects the electrical field and the electromigration. As Figure 5b shows, the *i*_local_ of the cathode is directly correlated to the cathode topography. During the first discharge (*t* = 5 h), the *i*_PR, cathode_ was averagely higher than *i*_VR, cathode_, especially in the junction region that a more than three times *i*_juction, cathode_ of *i*_VR, cathode_ was observed. For the anode surface, although the cathode topography heterogeneity was buffered by the porous separator, the *i*_PR, anode_ was still higher than *i*_VR, anode_ (Figure 5d). During the subsequent charging process (*t* = 15 h), although the *i*_*l*ocal, cathode_ was not changed much, the anode current density differences (*i*_PR, anode_ − *i*_VR, anode_) became larger, suggesting the current density heterogeneity on the anode is exacerbated upon the cycling. This heterogeneity of current density causes the uneven Li plating (Figure 5c). More Li plated on the PR than VR, causing the boundary of PR of Li moves much closer to the cathode than VR after one cycle (*t* = 20 h, Figure 5c). These results were consistent with the SEM characterization (Figure 4f). A similar current distribution and morphological evolution trend was confirmed on cathode and anode by using a more detailed model where both a porous cathode and polysulfide dissolution were considered (Figure S5, Supporting Information).^[^ ^26^, ^30^ ^]^

Based on the study of cathode topography and its effects on the Li anode, a S cathode and Li anode crosstalk mechanism was proposed. Given the high porosity and roughness of the S cathode, there are always high and low regions distributed locally along the electrode surface (Figure 5e). When the soft Li foil is used as the anode, the rough cathode easily creates indentations on the Li anode surface under pressure during cell assembly (Figure 5f), causing uneven contact between the two electrodes. These highly indented regions have better contact and thus smaller local resistance. While for the VR, loose contact, even small gaps, may exist locally, resulting a higher local resistance. When current is applied, electrochemical reactions will preferentially occur along the lower resistance regions, resulting uneven Li stripping/plating, as observed by SEM and XPS (Figure 4; Figure S4, Supporting Information). This leads to local Li volume expansion and pulverization, and thus electrolyte redistribution, which further exacerbates variation of local resistance and current density (Figure 5g). Under certain circumstances, Li dendrites may be formed at locations having extremely high local current densities.

Two factors play important roles in the ISC event. First, uneven contact is caused by the rough cathode topography at the initial state and its further exacerbation during cycling. As illustrated in Figure 5f, the uneven contact induces resistance variation across the electrode surface, as supported by the characterization of the cycled Li anode (Figure 4). The second factor is the electrolyte conditions. Under flood electrolyte conditions, the excess electrolyte would compensate for the local resistance variation to some extent by fully filling the gaps between the electrodes. However, under lean electrolyte conditions or with depletion of electrolyte, the effects of electrode topography are amplified due to the lack of electrolyte. This explains why the ISC occurs very early (during the fourth cycle) in the patterned electrode (Figure 3h). It should be noted that loss of available electrolyte is unavoidable in Li or any other rechargeable metal batteries because of the electrolyte side reactions and unavoidable electrode pulverization. This means for any electrolyte conditions, upon cell cycling, the rough electrodes will experience uneven electrolyte distribution eventually, and sooner for the rougher electrodes. In addition, those two factors become entangled during the cell cycling, forming high local current densities and causing dendric Li growth (Figure 5h). The early cell failure mechanism was studied with the cell cycling at moderate C rate. It should be noted that the observed mechanism is applicable for high‐loading S cathodes operated at both higher and lower current densities. The main difference observed is that a higher C rate leads to an earlier ISC, while a lower C rate has a later ISC (Figure S6, Supporting Information), which is related to the Li morphology and electrolyte depletion rate at different current densities.^[^ ^7c^ ^]^

**Figure 6:**
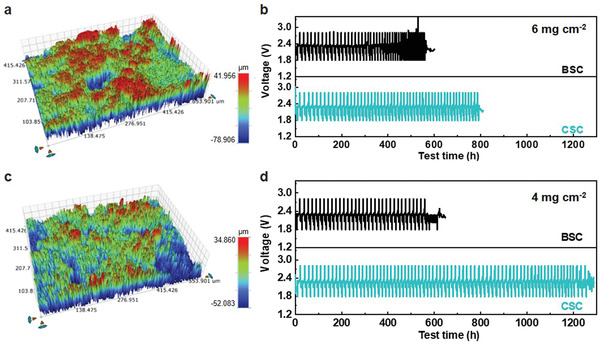
Optical profilometric images of CSC at S loading of a) 6 and c) 4 mg cm^−2^. Discharge and charge profiles of the BSC and CSC at S loading of b) 6 and d) 4 mg cm^−2^ in *E*/*S* = 4 mL g^−1^.

Based on our findings, any approaches that could homogenize cathode reactions and lower the variation of local currents would help to delay or eliminate an ISC event, thereby extending cell cycle life. Control of electrode topography is one of the most straightforward ways to accomplish this. To verify its effectiveness, a mild calendered electrode with a smoother surface was prepared. Only 10% compression was used to prepare the calendered S cathodes (CSC) and the measured average roughness was decreased by 20% compared to that of BSC (**Figure** **6**a). Without calendering, the BSC failed by ISC at 557 and 570 h (Figure 6b,d; Figure S7, Supporting Information) at a S loading of 6 and 4 mg cm^−2^, respectively. Promisingly, the CSC shows a significantly improved cycle life from 557 to 790 h at the same *E*/*S* of 4 mL g^−1^ (Figure 6b; Figure S8, Supporting Information). By lowering the areal loading from 6 to 4 mg cm^−2^, the cycle life improvement was even more profound, improving by 124% to 1251 h (Figure 6c,d). This further proves the importance of electrode topography on cell cycle life, where high electrode uniformity and low roughness are desired for Li batteries.

## 3 Conclusion

Early failure behaviors of Li–S cells were studied under practical conditions and the failures were ascribed to the crosstalk between the S cathode and Li anode. By using S electrodes with manipulated surface patterns, the impacts of cathode topography on electrochemical reaction, Li morphological evolution, and cell cycle life were investigated. It was proved that ISC is a key cause of early charge failure in realistic Li–S cells. We revealed that cathode topography has important impacts on the reaction uniformity of both the S cathode and Li anode, particularly under practical lean electrolyte conditions. Due to the softness nature of Li metal, the surface structures of S cathode are easily transferred to the Li anode, which affects electrolyte distribution and local resistances between the two electrodes. During cell operation, the inhomogeneous reactions happening on S cathodes are fully mirrored on the Li anodes and lead to local Li pulverization and expansion, thereby exacerbating the variation in local resistance. Ultimately, the entangled morphology evolution and electrolyte redistribution caused by the cathode and anode crosstalk lead to high localized current densities, inducing dendric Li growth and ISC. Control of cathode topography to homogenize resistances is demonstrated to be an effective way to suppress short circuit events and extend the cycle life of Li–S cells under practical conditions. The study sheds new light on electrode design to extend the cycle life of high‐energy Li–S and other rechargeable metal batteries.

## 4 Experimental Section

### Preparation of the S/IKB Composite

All the chemicals were used as received. The S host material, integrated Ketjenblack (IKB), was prepared based on the previously reported approach.^[^ ^17^ ^]^ In brief, Ketjenblack (KB, AkzoNobel) and citric acid (Sigma Aldrich) were mixed in water at a weight ratio of 1:1 and stirred at 60 °C for 2 h. Then ethylene glycol (Sigma Aldrich) was added into the dispersion at a ratio of ethylene glycol/citric acid = 2:1 mol mol^−1^ and stirred at 130 °C for 6 h. The mixture was dried overnight and calcined in a tube furnace at 800 °C for 10 h under an argon atmosphere. The obtained IKB was ground and sieved with 100‐mesh screens. To prepare the S/IKB composite, S powder (Alfa Aesar) was loaded into the pores of IKB via a melt‐impregnation process at 155 °C for 12 h. The S content in the S/IKB composite was 80 wt%.

### Preparation of the S Cathodes

Three kinds of S cathodes were prepared by the slurry‐coating method—a BSC, a PSC, and a CSC. The BSC was prepared by mixing S/IKB composite, C nanofibers (CNFs, Sigma Aldrich), and polyacrylic acid (Sigma Aldrich) with water as a solvent and *n*‐butanol (Sigma Aldrich) as an additive to form a uniform slurry. The weight ratio of active material, CNF, and binder was 8:1:1. Then the slurry was coated on a C‐coated Al foil (Guangzhou Nano New Material Technology Co., Ltd). The S cathode was dried at 60 °C under vacuum conditions for 12 h resulting in the BSC. To prepare the PSC, a coating process similar to the BSC process was used, but the coating was done twice. First, a dried BSC with a S loading of 4 mg cm^−2^ was prepared. Then Al mesh (80 µm thickness) as a template was put on the BSC. Slurry was coated on the Al mesh. After the whole piece was dried, the Al mesh was removed resulting in a PSC with a S loading of 6 mg cm^−2^. The CSC was prepared by calendering the BSC to 90% of its original thickness prior to use. The S loadings were controlled at 4 and 6 mg cm^−2^. Without annotation, a S loading 6 mg cm^−2^ was used. The S cathode had a thickness of 140 µm at 6 mg cm^−2^ of S loading.

### Assembly of the Coin Cell

The 2032‐type (MTI Corp.) coin cells were assembled in a glovebox (M. Braun) filled with argon with both O and moisture levels below 1 ppm. 250 µm Li chips (MTI Corp.) and 50 µm Li on Cu foil (China Energy Lithium Co., Ltd) were used as the Li anode for N/P 8.3 and N/P 1.7, respectively. Without annotation, 250 µm Li chips were used. The diameters of the cathode and anode were 12.7 and 15.9 mm, respectively. Celgard 2500 was used as the separator. The electrolyte used in this study was 1 m Li bis(trifluoromethanesulfonyl) imide (LiTFSI, Gotion) in 1,3‐dioxolane (DOL, Gotion) and 1,2‐dimethoxyethane (DME, Gotion) (1:1, v/v) with 0.3 m LiNO_3_ (Sigma Aldrich) as an additive. The *E*/*S* ratio is the ratio of the electrolyte volume (mL) to the S mass (g) in the cell assembly. For the reassembled cell, the cycled electrodes were assembled with fresh separator and electrolyte (*E*/*S =* 6 mL g^−1^) and tested in the coin cell.

### Electrochemical Test

The electrochemical performance of the coin cell was tested galvanostatically at 0.1 , 0.05, and 0.3 C (1 C = 1000 mA g^−1^) in a voltage range of 1.8–2.8 V on an Arbin BT2000 at 30 °C. Without annotation, 0.1 C was used. The charge/discharge specific capacity mentioned in this paper was calculated based on the S weight by excluding the C content. The reassembled coin cell was tested in 1.8–2.8 V at 0.02 C at 30 °C.

### Characterization

The cycled electrodes were characterized and electrochemically tested in a reassembled coin cell without undergoing a washing treatment. The used separators were washed in DOL and DME (1:1, v/v) mixed solvent three times to remove any residual polysulfide before characterization. Samples transferred to characterization instruments were sealed in air‐proof containers filled with argon to avoid air contamination. Observation of sample morphology and EDX spectroscopy analysis were performed using a JEOL JSM 7001F field emission SEM and a dual‐FIB/SEM (FEI Helios) system.

Surface roughness was characterized with an optical profilometer (Bruker ContourGT‐I) using white light interferometry. A 20× interferometric objective and 0.55× field‐of‐view lens were used to image a 0.22 mm^2^ area at a 0.1 nm vertical resolution. 3D height maps and line scans along the *X* and *Y* axes were obtained for five randomly selected areas on each electrode. An average surface roughness in terms of root mean square roughness (*R*_q_) was calculated and the results are summarized in Table S1, Supporting Information.

Samples with a size of 15 µm × 10 µm × 80 nm were prepared by FIB and then mounted on a Cu‐grid for Cryo‐EM in a liquid N_2_ environment to prevent the oxidation of Li. The as‐prepared sample was characterized by a 300 kV FEI Titan monochromated (S)TEM equipped with a Gatan Elsa cryo‐transfer holder. The EELS collection semi‐angle during the spectroscopy experiments was ≈45 mrad. The EELS spectra dispersion was 0.05 eV/channel with vertical binning at 130. The probe beam current was around 25 pA, and pixel dwell time was 0.001 s (integration time was 10 s). At first, EDX mapping was carried out as mentioned in Figure 2 to distinguish the two types of particles (T1 and T2). Then a region with low O concentration and low S concentration was selected and inspected at higher resolution.

## Conflict of Interest

The authors declare no conflict of interest.

## Supporting information

Supporting Information

## Data Availability

The data that support the findings of this study are available from the corresponding author upon reasonable request.
