# Supplementary material for: Early Failure of Lithium–Sulfur Batteries at Practical Conditions: Crosstalk between Sulfur Cathode and Lithium Anode
Source: Adv Sci (Weinh). 2022 May 7;9(21):2201640. doi: 10.1002/advs.202201640 (PMC9313511; doi:10.1002/advs.202201640)
Supplement: Supplementary file 1 — Supporting Information [file ADVS-9-2201640-s001.pdf]

Supporting Information

Early Failure of Lithium–sulfur Batteries at Practical Conditions: Crosstalk between Sulfur Cathode and Lithium Anode

*Lili Shi, Cassidy S. Anderson, Lubhani Mishra, Hong Qiao, Nathan Canfield, Yaobin Xu, Chengqi Wang, TaeJin Jang, Zhaoxin Yu, Shuo Feng, Phung M Le, Venkat R. Subramanian, Chongmin Wang, Jun Liu, Jie Xiao, and Dongping Lu**

Simulation of Li-S cell with rough cathode

Simplified model

This section details a simplified mathematical model[26] for the Li-S simulations to substantiate the findings for the crosstalk between the Li anode and S cathode. This model can be used to analyze Li plating/stripping at the Li anode during cycling. The effect of cathode topography on the Li electrodeposition for a uniform Li anode surface is studied.

The following model deals with the two-dimensional mass and charge transport in the separator with a binary electrolyte in the lithium/sulfur cell. The setup can be viewed as shown in Figure 4 (a) where the separator region being modeled is shown between the rough S cathode and pristine Li anode. The electrolyte consists of Li^+ ions which get stripped at anode-separator interface during discharge and undergo plating at the Li anode on the application of current while charging. The cell cycling has been modeled with the help of the following equations for the plating at the lithium anode.

For a binary electrolyte, the flux, N_i of each dissolved species i (i = 1 and 2 for Li^+ ion and A^- anion in the electrolyte, respectively) in terms of diffusion and migration can be written as below. The other ionic species for the soluble polysulfides in the electrolyte are not included in the present model.

$$N_i = -D_i \nabla c_i - z_i \frac{D_i}{RT} F c_i \nabla \phi_2 \tag{1}$$

Here c_i is the species concentration, ϕ_2 is the electrostatic potential, z_i is the electric charge on the ion, D_i is the effective diffusion coefficient of species i. F, R and T follow their standard

definitions for Faraday's constant, universal gas constant and absolute temperature, respectively.

For the Li metal anode, the charge transfer equation takes the form, $Li \rightleftharpoons Li^{+} + e^{-}$, thus, the ionic charge on the cation and anion, i.e., z_1 and z_2 are +1 and -1, respectively. The material balance of the ionic species can be written using the Nernst-Planck equation as:

$$\frac{\partial c_i}{\partial t} = -\nabla \cdot N_i - R_i \tag{2}$$

The rate of production (or consumption) of the precipitation/dissolution of species *i* given by R_i in Equation (2) is not considered due to the absence of any precipitation reactions in the domain of interest.

Also, the electroneutrality assumption is given below for a binary electrolyte:

$$\sum_i z_i c_i = 0 \tag{3}$$

Under the assumption for electroneutrality in Equation (3), the model can be simplified as $c_1 = c_2 = c$. On further mathematical simplification of these equations and assuming constant electrolyte diffusivity for both anion and cation, i.e., ($D_1 = D_2 = D$), we arrive at the following decoupled equations for the Li^+ concentration (c) and potential (ϕ_2) within the electrolyte:

$$\frac{\partial c}{\partial t} = \nabla \cdot \left(D \nabla c_i \right) \tag{4}$$

$$0 = \nabla \cdot \left(c \nabla \phi_2 \right) \tag{5}$$

At the electrode-electrolyte interfaces, the anionic flux is zero, i.e., $N_2 = 0$. Thus, the flux boundary conditions at the electrode surfaces can be interpreted in the following form:

$$\left. \begin{array}{l} N_1 = \dfrac{i_{BV}}{F} \\ N_2 = 0 \end{array} \right] \Rightarrow n.\left(\nabla c \right) = -\frac{i_{BV}}{2FD} + \frac{c v_n}{D}, \; n.\left(c \nabla \phi_2 \right) = -\frac{i_{BV}}{2FD}\left(\frac{RT}{F} \right) \tag{6}$$

where i_{BV} denotes the local current density due to the electrochemical reactions at the electrode-electrolyte interface governed by the modified Butler-Volmer kinetics in Equation (7).

η is the activation overpotential for the reaction, ϕ_1 is the solid phase electrode potential, and U_{eq} is the equilibrium potential. The solid phase potential at Li anode, $\phi_{1,anode}$, is set to be zero as a reference point for the potential and $U_{eq,anode}$ is assumed to be zero for the Li metal reaction

$$\begin{array}{l} i_{BV,anode} = 2 i_{0,anode} \left(\dfrac{c}{c_{0,anode}} \right)^{0.5} \sinh\left(\dfrac{\eta_{anode} F}{RT} \right), \quad \eta_{anode} = \cancel{\phi_{1,anode}} - \phi_2 - \cancel{U_{eq,anode}} \\ i_{BV,cathode} = -2 i_{0,cathode} \left(\dfrac{c}{c_{0,cathode}} \right)^{0.5} \sinh\left(\dfrac{\eta_{cathode} F}{RT} \right), \quad \eta_{cathode} = \phi_{1,cathode} - \phi_2 - U_{eq,cathode} \end{array} \tag{7}$$

The flux boundary condition at the moving anode interface can be modified with the addition of the advective flux term (= cv_n/D where v_n is the normal velocity at the moving interface) for mass conservation.[27] The remaining boundaries of the two-dimensional separator domain are considered to be insulated with a zero-flux boundary condition. Under galvanostatic operating conditions, a uniform current density, i_{app}, is applied at the cathode-electrolyte interface. This implies that the total applied current remains constant with time but the non-uniform topography at the rough S cathode will lead to a non-uniform local current density distribution along the cathode as well as anode surface. This requires the values for $\phi_{1,cathode}$ and $U_{eq,cathode}$ in the expression for activation overpotential. These have been calculated in a combined manner ($\phi_{1,cathode}$ - $U_{eq,cathode}$) by imposition of a constraint on the system in the form of an integral boundary condition. With the continuity of fluxes in the separator domain, this constraint is satisfied at the anode-electrolyte interface as well.[28]

$$\oint_{cathode} i_{local} ds = \oint_{cathode} i_{app} ds \quad (8)$$

where s represents a morphology function, $s(x,t)$ along the non-uniform electrode surfaces such that it captures the total arc length along any surface.

Finally, the velocity of the moving boundary at the anode is prescribed by the Faraday's law as[29]:

$$v_n = \frac{\partial s}{\partial t} = \frac{M_w}{\rho} \frac{i_{BV,anode}}{F} \quad (9)$$

where M_w and ρ represent the molar weight and density of Li metal, respectively.

The initial conditions for the system of equations are given as $c(x,y,0) = c_0$ and $s(x,0)$ for anode surface is 53 μm.

The system of partial-differential-algebraic equations (pDAEs), Eqs. (4-9), can be solved numerically by prescribing the appropriate boundary conditions and system constraints using the finite-element based solver, COMSOL Multiphysics. Careful numerical analysis has been performed for the mesh size and time stepping for a relative and absolute tolerances of 10^{-6} and 10^{-8}, respectively. The baseline model parameters used in the present simulations are enlisted in Table S2.

Detailed model

A numerical model was proposed by Kumaresan K. et al.[26a] to describe the electrochemistry process of Li-S battery cell, which considers the transport of multiple electrolyte species, and the cascade sulfur reactions. Zhang T. et al. [30] later retuned this model by reducing the bulk diffusivity from magnitude of 1×10^{-10} m^2 s^{-1} to 1×10^{-12} m^2 s^{-1} so the simulated discharge curve can agree with measurement using a 3.4 Ah Li-S pouch cell (OXIS Energy Ltd). In this present

work, the retuned model by Zhang T. et al. is adopted but the porosity of separator (0.45) and cathode (0.67) are replaced with our measurement.

Governing Equations:

The simulation domain consists of a porous separator, a liquid electrolyte region, and a porous cathode. The following Faradic reactions are considered in this simulation:

$$\frac{1}{2}S_8 + e^- \leftrightarrows \frac{1}{2}S_8^{2-} \tag{10}$$

$$\frac{3}{2}S_8^{2-} + e^- \leftrightarrows 2S_6^{2-} \tag{11}$$

$$S_6^{2-} + e^- \leftrightarrows \frac{3}{2}S_4^{2-} \tag{12}$$

$$\frac{1}{2}S_4^{2-} + e^- \leftrightarrows S_2^{2-} \tag{13}$$

$$\frac{1}{2}S_2^{2-} + e^- \leftrightarrows S^{2-} \tag{14}$$

In addition, the dissolution of S is represented by Eqs. (15):

$$S_{8(s)} \rightleftharpoons S_8 \tag{15}$$

Only the precipitation of Li_2S is considered in this study:

$$2Li^+ + S^{2-} \leftrightarrows Li_2S_{(s)} \tag{16}$$

It was assumed reactions (10) to (16) occur in the porous cathode. The transport of a total of 7 species are calculated by the Nernst-Plank equation:

$$\frac{\partial \varepsilon C_i}{\partial t} - \nabla \cdot \left(D_i^{eff} \nabla C_i + z_i \frac{D_i^{eff}}{RT} F C_i \nabla \phi_2 \right) = a_v \sum_j \frac{s_{i,j} i_j}{n_j F} - \sum_k \gamma_{i,k} k_k \alpha_k \left[\prod_i (C_i)^{\gamma_{i,k}} - K_{sp,k} \right] \tag{17A}$$

The second term in left hand side (LHS) of Equation (17A) represents the diffusion and migration of the *i-th* ionic species. The two terms in right hand side (RHS) describe the Faradic reaction and non-Faradic (dissolution or precipitation) respectively in order. The variables in Equation (17A) are summarized below:

ε porosity

C_i	concentration of i-th ionic species
t	time
D_i^{eff}	effective diffusivity, equal to $\epsilon^{1.5} D_i$ (Bruggeman assumption)
z_i	charge number of i-th ionic species
R	specific ideal gas constant
T	temperature
F	Faraday constant
ϕ_2	electrolyte phase potential
a_v	specific area of porous cathode
$s_{i,j}$	stoichiometric coefficient of species *i* in reaction *j*
i_j	superficial current density due to reaction *j*
n	number of electrons participating in reaction *j*
$\gamma_{i,k}$	number of moles of ionic species *i* in solid species *k* ($S_{8(s)}$ or $Li_2S_{(s)}$)
k_k	precipitation rate
α_k	volume fraction of precipitate *k* in porous cathode or separator
$K_{sp,k}$	Solubility product

The current density i_j in Equation (17A) is computed using the Butler-Volmer equation:

$$i_j = i_{0,j}\left[\prod_i \left(\frac{C_i}{C_{i,ref}}\right)^{p_{i,j}} \exp\left(\frac{0.5F}{RT}\eta_j\right) - \prod_i \left(\frac{C_i}{C_{i,ref}}\right)^{q_{i,j}} \exp\left(-\frac{0.5F}{RT}\eta_j\right)\right] \quad (18)$$

where

$i_{0,j}$	exchange current density for reaction *j*
$C_{i,ref}$	referenceconcentration of species *i* (assumed to be initial)
η_j	activation overpotential of reaction *j*
$p_{i,j}$	equals to $s_{i,j}$ for anodic species
$q_{i,j}$	equals to $-s_{i,j}$ for cathodic species

The summation of i_j constitutes the electrolyte phase current density:

$$\nabla \cdot \mathbf{i}_e = a_v \sum_j i_j \quad (19)$$

where a_v indicates the specific area of porous cathode.

The overpotential η_j in Equation (18) is calculated by

$$\eta_j = \phi_1 - \phi_2 - \left[U_j^\theta - \frac{RT}{n_j F}\ln\sum s_{i,j}\left(\frac{C_{i,\mathrm{ref}}}{10^3}\right)\right] \quad (20)$$

where:

ϕ_1	solid phase potential
U_j^θ	standard equilibrium potential at 1000 mol/m^3

The current in the battery cell satisfies continuity equation:

$$\nabla \cdot \mathbf{i}_e + \nabla \cdot \mathbf{i}_s = 0 \quad (21A)$$

where $\mathbf{i}_e$ and $\mathbf{i}_s$ are the liquid and solid phase current density, respectively. For the electrolyte domain $\mathbf{i}_s = 0$. The solid phase current density in the cathode satisfies Equation (22):

$$\mathbf{i}_s = -\sigma\nabla\phi_1 \quad (22)$$

The precipitation of Li_2S and dissolving of S will change the porosity of the cathode, which can be described by Equations (23) and (24):

$$\frac{\partial\varepsilon}{\partial t} = -\sum_k V_k R'_k \quad (23)$$

$$\frac{\partial\alpha_k}{\partial t} = -V_k R'_k \quad (24)$$

where V_k indicates the molar volume of solid species k. Accordingly, it was assumed the specific area of the porous cathode changes with porosity:

$$a_v = \left(\frac{\varepsilon}{\varepsilon_0}\right)^{1.5} \quad (25)$$

It was assumed there are no reactions in the electrolyte domain. Therefore, Equation (17A) can be used to describe the ionic transport by replacing the effective diffusivity with bulk diffusivity and removing the RHS terms:

$$\frac{\partial C_i}{\partial t} - \nabla \cdot \left(D_i\nabla C_i + z_i\frac{D_i}{RT}FC_i\nabla\phi_2\right) = 0 \quad (17B)$$

The current in liquid electrolyte domain is attributed only to the ionic flux $\boldsymbol{J}$:

$$\mathbf{i}_e = F\sum_i z_i\boldsymbol{J}_i = -F\sum_i z_i\left(D_i\nabla C_i + z_i\frac{D_i}{RT}FC_i\nabla\phi_2\right)\ , \quad (26)$$

and Equation (21A) reduces to

$$\nabla \cdot \mathbf{i}_e = 0 \tag{21B}$$

In addition, the electric neutrality should be satisfied:

$$\sum_i z_i C_i = 0 \tag{27}$$

Only the two non-Faradic reactions are considered in the porous separator. Accordingly, the ionic transport Equation (17A) can be reduced to Equation (17C):

$$\frac{\partial \varepsilon C_i}{\partial t} - \nabla \cdot \left(D_i^{eff} \nabla C_i + z_i \frac{D_i^{eff}}{RT} F C_i \nabla \phi_2 \right) = -\sum_k \gamma_{i,k} k_k \alpha_k \left[\prod_i (C_i)^{\gamma_{i,k}} - K_{sp,k} \right] \tag{17C}$$

In addition, the porous separator shares the same Equations (21B), (26) and (27) with the liquid electrolyte domain.

Boundary Conditions:

The ionic flux and the electrolyte phase current at the cathode collector is 0. Therefore, the solid phase current density equals to the applied current. The boundary conditions can be described by $\boldsymbol{J} = 0$, $\mathbf{i}_e = 0$, and $\mathbf{i}_s = I_{app}$.

At the interface between porous cathode and liquid electrolyte, the solid phase current density becomes 0, and the electrolyte current density and ionic flux is continuous. These boundary conditions are described as $\boldsymbol{J}^+ = \boldsymbol{J}^-$, $\boldsymbol{i}_e^+ = \boldsymbol{i}_e^-$ and $\mathbf{i}_s = 0$. These boundary conditions also apply to the separator-to-electrolyte interface.

For the anode-to-separator interface, the solid phase current density $\mathbf{i}_s$ and solid potential ϕ_1 are 0. The ionic flux is 0 except for Li^+. The Li^+ flux is given by: $\boldsymbol{J}_{Li^+} = \frac{i_{Li}}{F}$ And the corresponding electrolyte phase current density is given by: $\boldsymbol{i}_e = \mathrm{F}\boldsymbol{J}_{Li^+}$.

The application of above governing equations and boundary conditions to the battery cell can be illustrated by Figure S9. The values of modeling coefficients and constants can be found in Zhang T. et al.[30], except that the porosities of separator and cathode are changed to 0.45 and 0.68, respectively per measurement.

Table S1 The average electrode roughness of PSC, BSC, CSC with a S loading (L_s) of 6 mg cm^{-2}, and CSC with a L_s of 4 mg cm^{-2}.

Electrode	PSC	BSC	CSC_L_s 6	CSC_L_s 4
Average roughness (R_q, µm)	30	24	19	12

Table S2 Parameters for the simplified model simulation.

Symbols	Parameters	Values	Units
L_x	Domain length in *x*	551	μm
$i_{app,0.1C}$	Applied current density	6	A/m^2
$i_{0,anode}$	Exchange current density	20	A/m^2
$i_{0,cathode}$	Exchange current density	1.972	A/m^2
c_0	Initial concentration	1300	mol/m^3
$c_{0,anode}$	Reference anode concentration	1001	mol/m^3
$c_{0,cathode}$	Reference cathode concentration	5e-6	mol/m^3
D	Diffusion coefficient	1e-10	m^2/s
M_w	Molar mass of Lithium	6.941	g/mol
ρ	Density of Lithium	0.534	g/cm^3
T	Operating temperature	298	K
R	Gas Constant	8.314	J/mol.K
F	Faraday's Constant	96487	C/mol

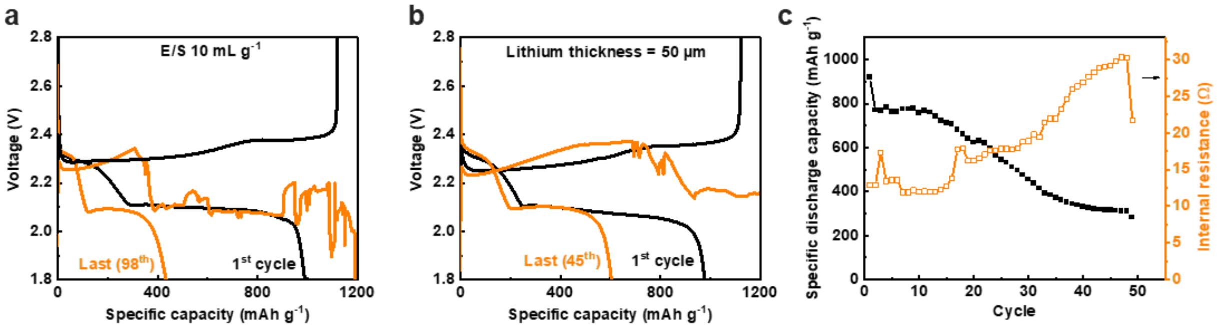

Figure S1 Discharge and charge profiles for the last cycles of the BSC under different conditions: (a) at E/S 10 mL g^{-1} and with 250 μm Li and (b) at E/S 10 mL g^{-1} and with 50 μm Li. (c) IR evolution of BSC upon cell cycling at E/S 4 mL g^{-1}.

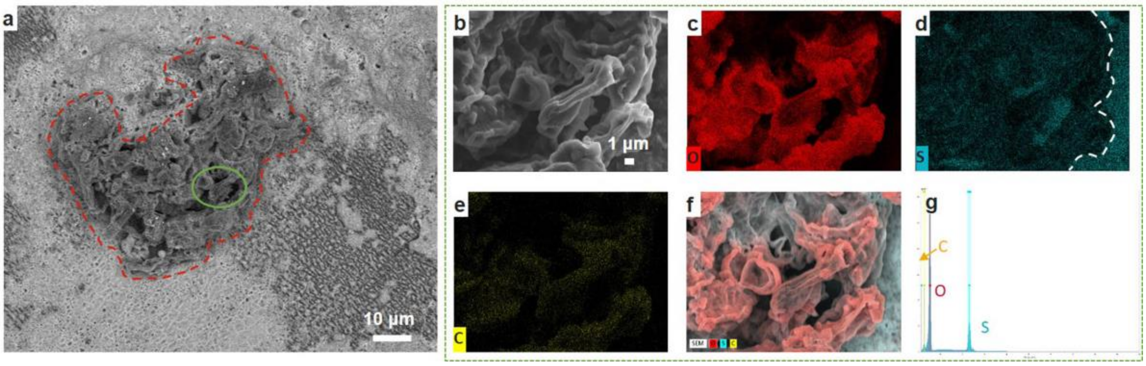

Figure S2 (a) BSE image of a T1 particle (green circle) in Figure 2b. (b) Higher-resolution SEM image of the T1 particle in the green circle of (a). (c-d) EDX mapping of (b). Red is O, blue is S, yellow is C. (b)-(e) are combined in (f). (g) EDX spectrum of (b). Only O, S, and C are detected.

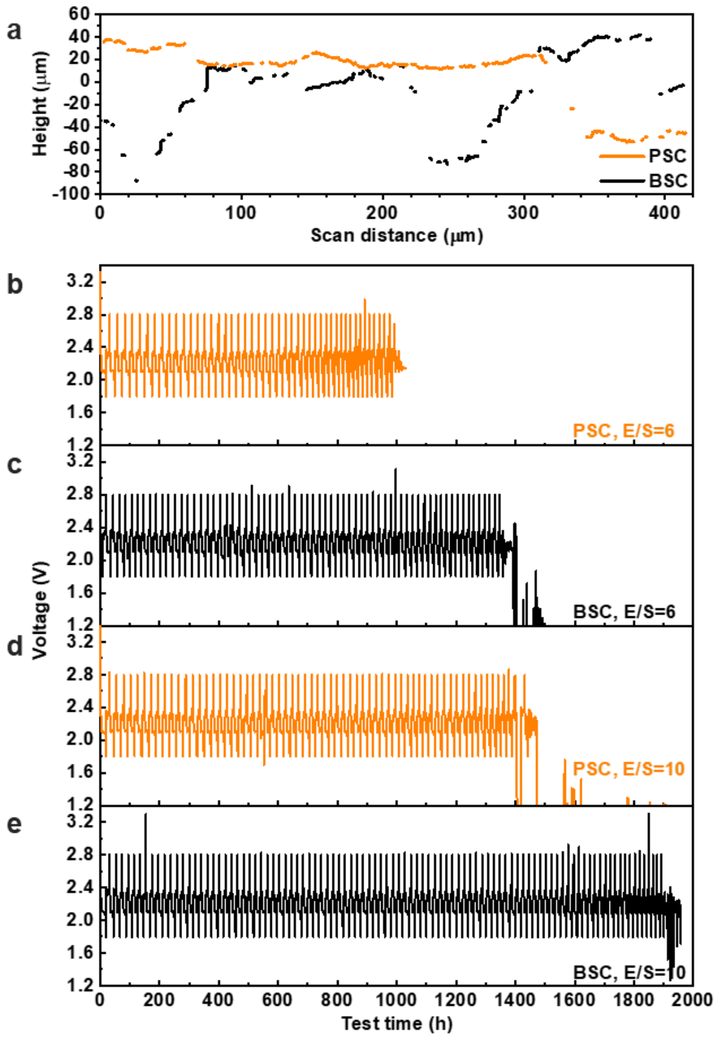

Figure S3 (a) Y line-scan profiles of PSC and BSC. The discharge and charge profiles of PSC (b and d) and BSC (c and e) at different electrolyte amounts: E/S 6 mL g^{-1} (b and c) and E/S 10 mL g^{-1} (d and e).

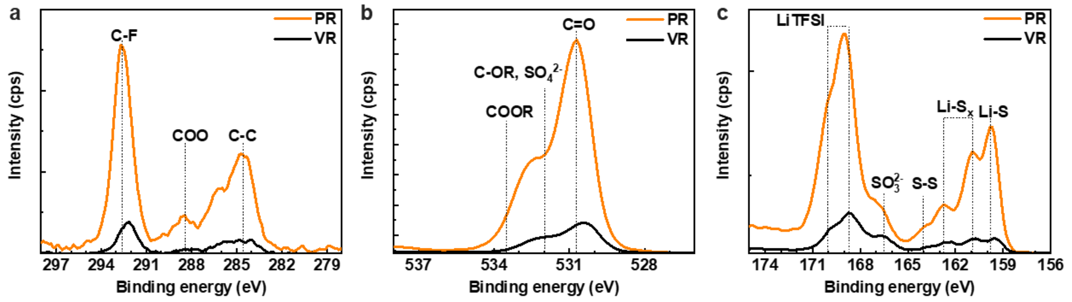

Figure S4 XPS C 1s (a), O 1s (b), and S 2p (c) spectra of the cycled Li surface with PSC. The orange spectra are from PR. The black spectra are from VR.

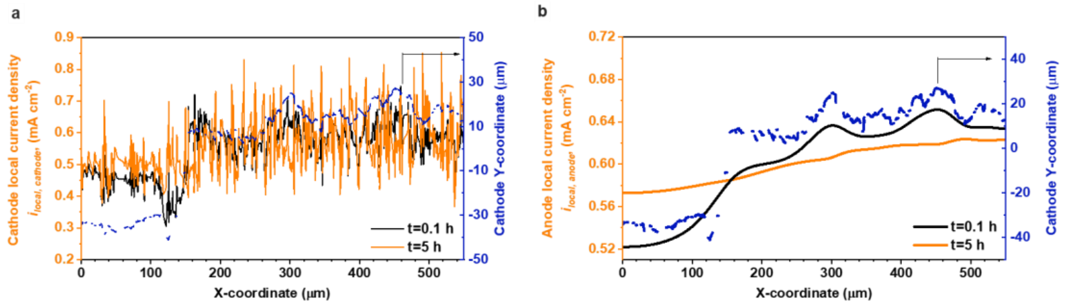

Figure S5 Simulated local current density distributions on the cathode (a) and anode (b) surface using detailed model.

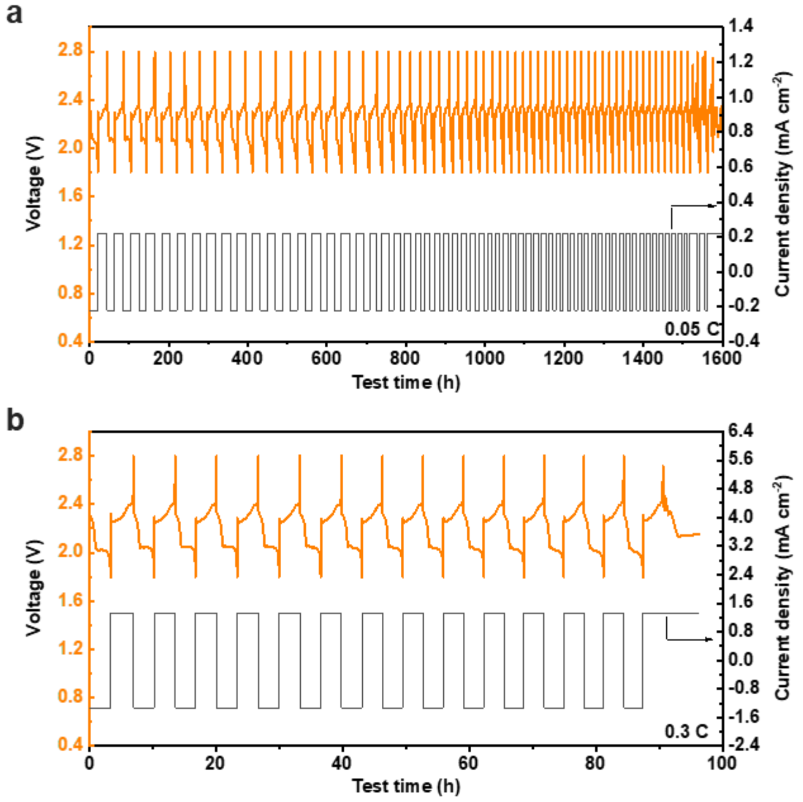

Figure S6 Discharge and charge profiles of BSC at different current densities: (a) at a S loading of 4 mg cm^{-2}, 0.05 C; (b) at a S loading of 4 mg cm^{-2}, 0.3 C.

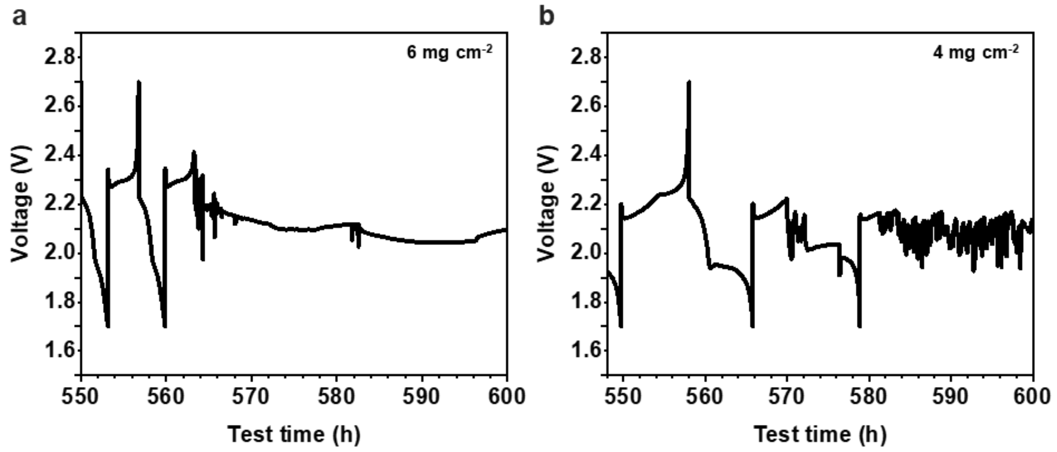

Figure S7 Zoomed-in charging profiles of BSC in the last cycles in Figure 6: (a) at a S loading of 6 mg cm^{-2}, (b) at a S loading of 4 mg cm^{-2}.

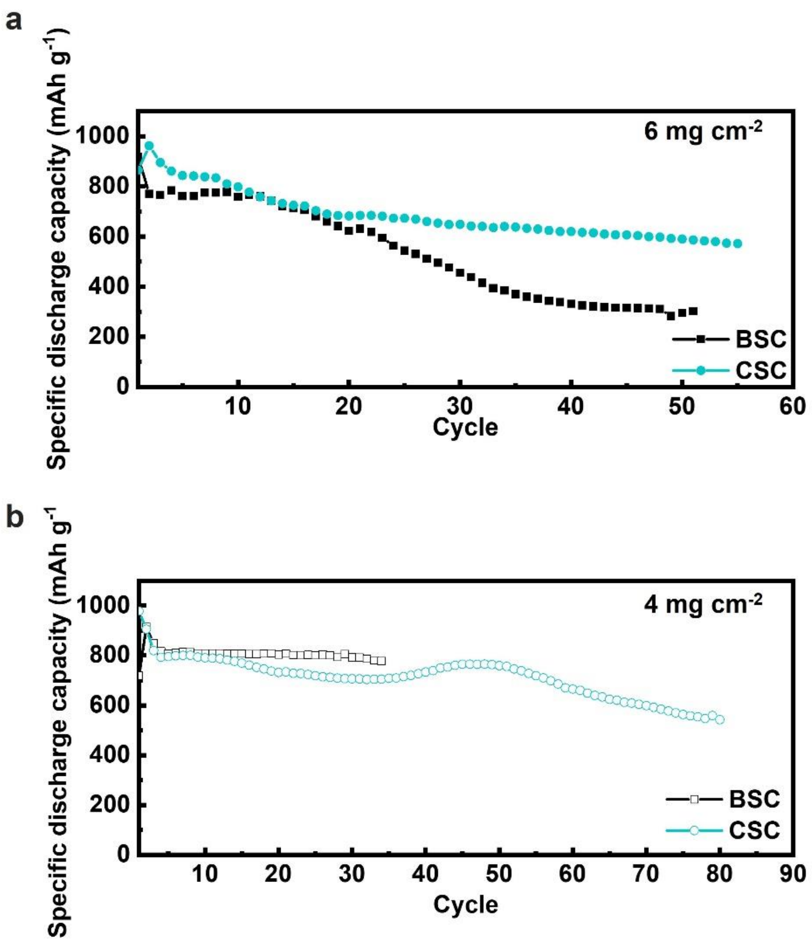

Figure S8 Cycle performance of BSC and CSC in Figure 6, (a) L_s = 6 mg cm^{-2}, (b) L_s = 4 mg cm^{-2}.

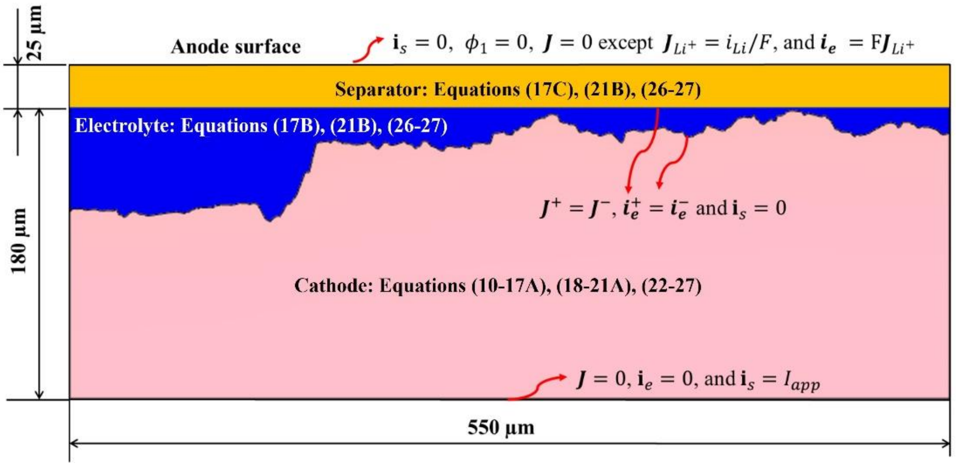

Figure S9 Geometry and equations of the detailed model.
